# Epidemiology and source of infection in cancer patients with febrile neutropenia: an experience from a developing country

**DOI:** 10.1186/s12879-023-08058-6

**Published:** 2023-02-22

**Authors:** Nagham Joudeh, Elana Sawafta, Adham Abu Taha, Majd Hamed Allah, Riad Amer, Razan Y. Odeh, Husam Salameh, Ali Sabateen, Banan M. Aiesh, Sa’ed H. Zyoud

**Affiliations:** 1 Department of Medicine, College of Medicine and Health Sciences, An-Najah National University, Nablus, 44839 Palestine; 2 Department of Biomedical Sciences, Faculty of Medicine and Health Sciences, An-Najah National University, Nablus, 44839 Palestine; 3 Department of Pathology, An-Najah National University Hospital, Nablus, 44839 Palestine; 4 Department of Hematology and Oncology, An-Najah National University Hospital, Nablus, 44839 Palestine; 5 Infection Control Department, An-Najah National University Hospital, Nablus, 44839 Palestine; 6 Department of Clinical and Community Pharmacy, College of Medicine and Health Sciences, An-Najah National University, Nablus, 44839 Palestine; 7 Poison Control and Drug Information Center (PCDIC), College of Medicine and Health Sciences, An-Najah National University, Nablus, 44839 Palestine; 8 Clinical Research Center, An-Najah National University Hospital, Nablus, 44839 Palestine

**Keywords:** Antimicrobial resistance, Febrile neutropenia, Cancer patients

## Abstract

**Background:**

Febrile neutropenia (FN) is a life-threatening complication that predisposes cancer patients to serious infections. This study aims to describe the epidemiology and source of infection in cancer patients with FN in a tertiary care hospital.

**Methods:**

A hospital-based retrospective study was conducted in a large tertiary care hospital from January 2020 to December 2021. Data on cancer patients with FN were collected from the hospital information system.

**Results:**

150 cancer patients with FN were identified during the study period. Most patients were males (98; 65.3%), and the mean age of participants was 42.2 ± 16.0 years. Most patients (127; 84.7%) had hematologic malignancies, and acute myeloid leukemia was the most common diagnosis (42; 28%), followed by acute lymphocytic leukemia (28; 18.7%) and Hodgkin’s lymphoma (20; 13.3%). Fifty-four (36%) patients had a median Multinational Association for Supportive Care in Cancer (MASCC) scores greater than 21. Regarding the outcome, nine (6%) died, and 141(94%) were discharged. The focus of fever was unknown in most patients (108; 72%). Among the known origins of fever were colitis (12; 8%), pneumonia (8; 5.3%), cellulitis (6; 4%), bloodstream infections (7; 4.6%), perianal abscess (2; 1.3%) and others. The median duration of fever was two days, and the median duration of neutropenia was seven days. Sixty-three (42%) patients had infections: 56 (73.3%) were bacterial, four (2.6%) were viral, two (1%) were fungal and 1 (0.7%) was parasitic. Among the bacterial causes, 50 cases (89.2%) were culture-positive. Among the culture-positive cases, 34 (68%) were gram-positive and 22 (44%) were gram-negative. The most frequent gram-positive bacteria were *E. faecalis* (9; 18% of culture-positive cases), and the most frequent gram-negative organisms were *Klebsiella pneumoniae* (5; 10%). Levofloxacin was the most commonly used prophylactic antibiotic (23; 15.33%), followed by acyclovir (1610.7%) and fluconazole in 15 patients (10%). Amikacin was the most popular empiric therapy, followed by piperacillin/tazobactam (74; 49.3%), ceftazidime (70; 46.7%), and vancomycin (63; 42%). One-third of *E. faecalis* isolates were resistant to ampicillin. Approximately two-thirds of *Klebsiella pneumoniae* isolates were resistant to piperacillin/tazobactam and ceftazidime. Amikacin resistance was proven in 20% of isolates.

**Conclusions:**

The majority of patients suffered from hematologic malignancies. Less than half of the patients had infections, and the majority were bacterial. Gram-positive bacteria comprised two-thirds of cases. Therefore, empiric therapy was appropriate and in accordance with the antibiogram of the isolated bacteria.

## Background

Cancer is one of the most diseases that researchers have studied for several decades. It is an important cause of death, with a prevalence of > 10 million deaths per year [1]. Cancer is abnormal body cells that divide without control and can spread to nearby tissues. Human cells that have been attracted by cancer and, to some extent, changed into pathogenic organisms or tumor-forming components are destructive agents [2, 3]. Traditionally, cancer patients were treated with surgery, chemotherapy, radiation, immunotherapy, targeted therapy, or hormonal therapy [4].

Chemotherapy is approved to be curative for some types of cancer; otherwise, it could be considered an effective treatment for most cancers [5]. Chemotherapy agents have a cytotoxic impact on cancer cells. However, because these activities are not selectively toxic, they can be lethal to normal body cells in addition to the impact on cancer cells. The side effect is the damage caused by the agents to normal cells. Side effects decrease the quality of patient life and complicate the treatment [6].

Many side effects have been reported. The most disturbing side effects of chemotherapy are nausea, hair loss, and vomiting [7]. An important and serious side effect of cytotoxic chemotherapy is febrile neutropenia (FN) which is the first and could be the only sign of infection in cancer patients [8].

The main function of neutrophils is to provide a host defence mechanism against infection, particularly bacterial and fungal infections [9]. Although fever is an indicating sign, patients can come with normal temperature or even hypothermia [9]. The average number of complications related to FN is roughly 25% to 30%, and the mortality rate is as high as 11% in some groups [10]. In addition, hospital and admission mortality could reach 50% in severe and septic shock [9].

Febrile neutropenia is a life-threatening complication that predisposes cancer patients with cancer to serious infections and reduces their intake of optimal therapeutic doses of chemotherapy [10]. The incidence of bacteremia with gram-negative organisms is more than with gram-positive. *Enterobacteriaceae* sp. is the most predominant, followed by *P. aeruginosa* and other Gram-negative. Resistant pathogens, unfortunately, have progressed because of the use of prophylactic antibiotics such as *extended-spectrum beta-lactamase* (ESBL). The most important causes of bacteremia among gram-positive organisms are *Staphylococcus aureus*, including *Methicillin-resistant Staphylococcus aureus (MRSA),* coagulase-negative *Staphylococcus*, *Streptococcus* of the viridans group, and *Enterococci*, especially *vancomycin-resistant* *Enterococcus* (VRE). Anaerobic bacteria can cause polymicrobial infections in patients with abdominal surgery. Fungal infections are not as common as bacterial infections, but when they occur, *Aspergillus sp*. and *Candida sp*. [11].

The Multinational Association of Supportive Care of Cancer (MASCC) score assesses risk among febrile neutropenic cancer [12]. Patients with a high MASCC score were deemed low risk after a blood culture was obtained. They planned to be treated as outpatients and usually given a dose of intravenous antibiotics, then can be treated with an oral regimen [8]. Increasing resistance to antimicrobial agents is a recurrence and a big problem in days, especially those applied to *ESBL*, *Pseudomonas aeruginosa*, *MRSA*, *VRE*, and even *carbapenemase-producing Enterobacteriaceae* *(CPE)* [13].

Many studies have shown that febrile neutropenia patients who have developed sepsis or colonized with multidrug-resistant pathogens are considered a selected subgroup that needs to be treated only with carbapenems or antibiotic combination as primary empirical therapy [14]. Although multidrug-resistant pathogens continue to appear, new antimicrobial agents under development are rare. As a result, the logical use of antibiotics based on antibiotic direction rules is critical [14]. In addition, using one class of antibacterial agents broadly can enhance multiclass drug resistance [15].

Few studies have been conducted on the use of antibiotics in hospitals in Palestine [16–18], and none have looked at FN. Therefore, this study aims to determine antimicrobial resistance among cancer patients who developed FN from chemotherapy in a tertiary academic hospital in Palestine. We believe this study will help the scientific community better approach and decrease the morbidity and mortality of FN in cancer patients. The results will give better knowledge about the selected agents’ antimicrobial resistance and sensitivity pattern. Furthermore, this study will help determine the best antibiotic regimen for those patients. It can also open opportunities for new research on this condition or change the current treatment guidelines.

## Methods

### Study design

A retrospective cohort study was conducted from 01/2020 to 10/2021 in the oncology center at An-Najah National University Hospital.

### Setting

This study was carried out in Nablus, Palestine, in the main tertiary hospital, An-Najah National University Hospital, which provided services to a total population of over 300,000 people in the north of the West Bank district.

### Study population

Cancer patients who developed FN in the northern West Bank, in the main large tertiary hospital, An-Najah National University Hospital, were included in our study.

### Sample size

One hundred and fifty patients who were recorded with neutropenic fever at An-Najah National University Hospital were our sample.

### Inclusion and exclusion criteria

#### Inclusion criteria

Male or female cancer patients with neutropenic fever due to chemotherapy were included in this study.

#### Exclusion criteria

1. Patients who have received cancer treatment other than chemotherapy.
2. Pediatric patients.

### Data collection instrument

Data were collected from patient's medical records at NNUH and filled in the data collection form. The data collection form included the following sections:

“Background” section contains data related to patient’s age, sex, previous comorbidities, and duration of fever and neutropenia.

“Methods” section contains data related to the type of cancer.

“Results” section contains data related to the outcome of patients with FN.

“Discussion” section contains data related to microorganisms isolated from febrile neutropenic patients.

“Strengths and limitations” section contains data related to antimicrobial resistance.

“Conclusion” section contains data related to antimicrobials used as prophylactic, empiric, or definitive.

### Operational definitions

A patient is considered to have a neutropenic fever if they have a one-time oral temperature of greater than 38.3 °C (approximately 100.9°F) or a sustained temperature of greater than 38 °C (100.4°F) for 1 h. Additionally, the patient must have an absolute neutrophil count of less than 500 cells/μL or an absolute neutrophil count that is expected to decrease to less than 500 cells/μL within the next 48 h [8].

### Microbial identification and antimicrobial susceptibility

Samples of different origins were cultured and bacterial identification and antibiotic susceptibility were determined by using the VITEK^®^ 2 Compact system (Automated instrument for ID/AST testing) (bioMérieux, Marcy-l’Étoile, France) based on the Clinical and Laboratory Standards Institute latest edition [19]. VITEK-2 identification cards were used for gram-positive, gram-negative bacteria and yeast. VITEK-2 AST-cards were used for performing antimicrobial susceptibility (antibiotics and antifungal susceptibility). The presence of *C. difficile* was tested in fecal specimens by using a qualitative immunoassay for detection of *Clostridium difficile* Glutamate Dehydrogenase (GDH), Toxin A and Toxin B (CoproStrip™ *C. difficile* Toxin A + B and Glutamate Dehydrogenase (GDH) from Savyon Diagnostics [20].

### Ethical approval

The An-Najah National University institutional review board (IRB) approved the research. In addition, approval to access data was obtained from An-Najah National University Hospital.

### Statistical analysis

The data were coded, categorized, and entered into the Statistical Package for Social Science (IBM-SPSS), version 21.0. Descriptive statistics [e.g., frequency, percentage, mean, standard deviation, median, interquartile range (IQR)] illustrate the sociodemographic and clinical data.

## Results

### Demographic and clinical characteristics of the study population

One hundred fifty patients with FN were found during the study period, with a mean age ± standard deviation (SD) of 40.2 ± 16. Among them, 65.3% were males. However, 31.3% of the patients had central venous catheters.

Regarding comorbidities, 14% of the patients had diabetes mellitus, 10% had hypertension, 5.3% had ischemic heart disease, and 3.3% had hepatitis B.

Regarding the focus of the fever, the study found that 72% of the patients had not identified the focus of their fever. However, 8% of the patients had colitis, followed by 5.3% had pneumonia, Furthermore, 4% had cellulitis. An important focus of the fever was bloodstream infection, with 4.6% of patients. Among seven bloodstream infections, three were related to Central Venous Catheters (CVC), three were secondary to other sources, and a peripheral blood infection caused one. The patients in our study had a median duration of fever- interquartile range (IQR) of 2 (1–4) days and a median duration of neutropenia (IQR) of 7 (4–14) days.

About 32.7% had positive cultures. Among the positive cultures, 26.5% had a polymicrobial infection that involved two or more bacterial pathogens, as shown in Table 1.

**Table 1:** Demographic and clinical characteristics of patients with febrile neutropenia

Variable	n (%)
Age (years), mean ± SD	40.2 ± 16
Sex	
Male	98 (65.3)
Female	52 (34.7)
Presence of CVC	
Yes	47 (31.3)
No	103 (68.7)
Comorbidities	
Diabetes mellitus	21 (14)
Hypertension	15 (10)
COPD	4 (2.6)
Ischemic heart disease	8 (5.3)
Osteoarthritis	1 (0.7)
Depression	1 (0.7)
Hepatitis B	5 (3.3)
PCOS	1 (0.7)
CHF	1 (0.7)
Epilepsy	1 (0.7)
Atrial fibrillation	1 (1.3)
Other arrythmias	2 (1.3)
Thrombophilia	1 (0.7)
Hypothyroidism	1 (0.7)
Ulcerative colitis	1 (0.7)
Liver cirrhosis	1 (0.7)
Primary sclerosing cholangitis	1 (0.7)
Focus of fever	
None identified	108 (72)
BSI	7 (4.6)
Cellulitis	6 (4)
Osteomyelitis	1 (0.7)
Colitis	12 (8)
Pneumonia	8 (5.3)
Bronchitis	1 (0.7)
Meningoencephalitis	1 (0.7)
Buccal ulcer	1 (0.7)
Perianal abscess	2 (1.3)
Mucositis	1 (0.7)
Jaw infection	1 (0.7)
Intraabdominal infection	1 (0.7)
Duration of fever (days), median and IQR	2 (1–4)
Duration of neutropenia (days), median and IQR	7 (4–14)
Culture positive	
Yes	49 (32.7)
No	101 (67.3)
Polymicrobial infection (among positive cultures)	
Yes	13 (26.5)
No	34 (73.5)

CVC, central venous catheters; SD, slandered deviation; COPD, chronic obstructive pulmonary disease; CHF, congestive heart failure; BSI, bloodstream infection; IQR, interquartile range

### Types of cancer in febrile neutropenic patients

Among the patients included in our study, acute myelogenous leukemia (AML) was found to be the most common type of cancer (28%), followed by acute lymphoblastic leukemia (ALL) (18.7%), then patients with Hodgkin lymphoma (13.3%) as shown in Table 2.

**Table 2:** Types of cancer in febrile neutropenic patients

Type of CA	n (%)
Diffuse large B-cell lymphoma	6 (4)
B-cell lymphoma (other)	10 (6.7)
Follicular lymphoma	1 (0.7)
Hodgkin’s lymphoma	20 (13.3)
Burkitt’s lymphoma	3 (2)
Mantle-cell lymphoma	1 (0.7)
Gastric lymphoma	1 (0.7)
Multiple myeloma	12 (8)
Hairy-cell leukemia	3 (2)
AML	42 (28)
ALL	28 (18.7)
CLL	3 (2)
Lung adenocarcinoma	1 (0.7)
Small cell lung cancer	1 (0.7)
Rectal cancer	1 (0.7)
Colon Adenocarcinoma	2 (1.3)
Cholangiocarcinoma	1 (0.7)
Gastric adenocarcinoma	3 (2)
Pancreatic adenocarcinoma	1 (0.7)
Retroperitoneal liposarcoma	2 (1.3)
Maxillary sinus adenocarcinoma	1 (0.7)
Brain cancer	1 (0.7)
Ewing sarcoma	3 (2)
Osteosarcoma	1 (0.7)
Testicular cancer	1 (0.7)
Breast cancer	1 (0.7)

AML, acute myeloid leukemia; ALL, acute lymphocytic leukemia; CLL, chronic lymphocytic leukemia

### Outcomes of patients with FN

As a complication of FN, 9.3% of the patients developed shock. Furthermore, 9.3% were admitted to the intensive care unit (ICU) during FN. According to renal dysfunction, 16.7% of the patients developed kidney injury, and 7.3% developed a liver injury.

Regarding the MASCC score, the median was calculated to be 20 with an IQR of (18–22). The score was less than 21 in 64% of the patients. During the hospital stay, 13.3% of the patients needed O2 therapy. Some patients are placed under mechanical ventilation (5.3%). In the end, about 6% of patients died, as shown in Table 3.

**Table 3:** Outcomes of febrile neutropenic patients

Variable	n (%)
Need for oxygen therapy	
Yes	20 (13.3
No	130 (86.7)
Mechanical ventilation	
Yes	8 (5.3)
No	142 (94.7)
Shock	
Yes	14 (9.3)
No	136 (90.7)
ICU admission	
Yes	14 (9.3)
No	136 (90.7)
Renal dysfunction	
Yes	25 (16.7)
No	125 (83.3)
Hepatic dysfunction	
Yes	11 (7.3)
No	139 (92.7)
MASCC score, median and IQR	20 (18–22)
MASCC score category	
Less than 21	96 (64)
Greater than or equal to 21	54 (36)
Outcome	
Discharged	141 (94)
Died	9 (6)

ICU, intensive care unit; MASCC, The Multinational Association for Supportive Care in Cancer, IQR, interquartile range

### Microbial profiles

Among the 49 positive cultures, 63 microorganisms were isolated. Gram-positive manifested in about 54% of the cases. However, about 35% were gram-negative. Fungal infections represented 3.2% of the cases. Viral infections had a rate of 6.3% of the cases.

Among gram-positive isolates, *E. faecalis* (14.3%) was predominant. The gram-negative isolates most frequently were *K. pneumonia* (7.9%), followed by *P. aeruginosa* (6.3%). Next, *E. coli* were divided into *non-extended-spectrum beta-lactamase (ESBL)* at 3.2% and *E. coli* at 4.8%. These were followed *by A. baumannii, with a percentage of* 4.8%.

Regarding fungal isolates, they were presented by *C. tropicalis* and *C. glabrata,* with 1.6% for each among all isolated cultures. *E. histolytica* made the protozoal isolates 1.6%. Coronavirus Disease (COVID-19) among viral isolates ranked at 6.3% of febrile neutropenic cases, as shown in Table 4.

**Table 4:** Organisms isolated from febrile neutropenic patients

Organism	n (%)
Gram-positive bacteria	34 (53.96)
*Enterococcus fecalis*	9 (14.3)
*Clostridioides difficile*	6 (9.5)
*Lactobacilli*	3 (4.8)
*Staphylococcus epidermidis*	2 (3.2)
*Streptococcus oralis*	2 (3.2)
*Corynebacteria*	2 (3.2)
*MRSA*	1 (1.6)
*MSSA*	1 (1.6)
*Staphylococcus haemolyticus*	1 (1.6)
*Staphylococcus cohnii*	1 (1.6)
*Staphylococcus capitis*	1 (1.6)
*Streptococcus agalactiae*	1 (1.6)
*Streptococcus gallolyticus*	1 (1.6)
*Streptococcus parasangunis*	1 (1.6)
*Micrococcus luteus*	1 (1.6)
*VRE*	1 (1.6)
Gram-negative bacteria	22 (34.92)
*Klebsiella pneumoniae*	5 (7.9)
*Pseudomonas aeruginosa*	4 (6.3)
*ESBL-E. coli*	3 (4.8)
*Acinetobacter baumannii*	3 (4.8)
*Non-ESBL E. coli*	2 (3.2)
*Neisseria sicca*	2 (3.2)
*Sphingomonas paucimobilis*	1 (1.6)
*Escherichia fergusonii*	1 (1.6)
*Pseudomonas stutzeri*	1 (1.6)
Fungi	2 (3.2)
*Candida tropicalis*	1 (1.6)
*Candida glabrata*	1 (1.6)
Protozoa	1 (1.6)
*Entamoeba histolytica*	1 (1.6)
Viruses	4 (6.3)
Coronavirus	4 (6.3)
Total	63 (100)

MRSA, methicillin-resistant *Staphylococcus aureus*; MSSA, methicillin-sensitive *Staphylococcus aureus*; VRE, vancomycin-resistant *Enterococcus;* ESBL, *extended-spectrum beta-lactamase*

### Antimicrobials used before and after culture

Levofloxacin was the most commonly used prophylactic antibiotic (15.33%). Regarding therapeutic regimens, amikacin was the most common empiric therapy (82.7%), followed by piperacillin/tazobactam (49.33%), ceftazidime (46.7%), and vancomycin (42%).

In terms of culture-guided antibiotics, amikacin was the agent most frequently used (46%), followed by vancomycin (38.7%), then ceftazidime (35.33%), as shown in Tables 5, 6, and 7.

**Table 5:** Prophylactic antimicrobials used

Antimicrobials	N (%)
Levofloxacin	23 (15.33)
Ciprofloxacin	2 (1.33)
Amoxicillin–clavulanate	2 (1.33)
Clarithromycin	1 (0.7)
Trimethoprim–sulfamethoxazole	1 (0.7)

**Table 6:** Empiric antimicrobials used

Antimicrobials	N (%)
Amikacin	124 (82.7)
Piperacillin/Tazobactam	74 (49.33)
Ceftazidime	70 (46.7)
Vancomycin	63 (42)
Metronidazole	8 (5.33)
Meropenem	7 (4.7)
Fluconazole	6 (4)
Levofloxacin	5 (3.33)
Ciprofloxacin	5 (3.33)
Amphotericin B	3 (2)
Tigecycline	2 (1.33)
Colistin	2 (1.33)
Ceftriaxone	1 (0.7)
Azithromycin	1 (0.7)
Ampicillin	1 (0.7)

**Table 7:** Definitive antimicrobial therapy

Antimicrobials	N (%)
Amikacin	69 (46)
Vancomycin	58 (38.7)
Ceftazidime	53 (35.33)
Piperacillin/Tazobactam	51 (34)
Meropenem	26 (17.33)
Ciprofloxacin	17 (11.33)
Levofloxacin	11 (7.33)
Tigecycline	11 (7.33)
Fluconazole	5 (3.33)
Amphotericin B	4 (2.7)
Metronidazole	3 (2)
Ceftriaxone	2 (1.33)
Cefuroxime	1 (0.7)
Gentamicin	1 (0.7)
Trimethoprim-sulfamethoxazole	1 (0.7)
Colistin	1 (0.7)

### Antimicrobial resistance of gram-positive isolates

The most frequently isolated gram-positive bacteria, 33.3%, were resistant to ampicillin and 42.9% of isolates were resistant to piperacillin/tazobactam. Thirty percent of the reported *Enterococcus* were resistant to vancomycin and interpreted as Vancomycin-Resistant *Enterococcus* (VRE).

Among *lactobacilli*, 50% were resistant to ampicillin, and 50% were resistant to amoxicillin clavulanate. In contrast, all isolates were resistant to TMP/SMX. A lower percentage (33%) were resistant to ceftriaxone. One hundred percent of *lactobacilli* were sensitive to piperacillin/tazobactam, imipenem, and cefotaxime.

Among *S. auerus* isolates, one isolate was *MRSA* sensitive to TMP/SMX. The other isolate was *MSSA* and was found to be resistant to benzylpenicillin without resistance to amoxicillin-clavulanate or cefuroxime, ciprofloxacin, levofloxacin, moxifloxacin, and gentamicin or TMP/SMX cephalosporins. All isolates of *S. epidermidis* were resistant to amoxicillin-clavulanate, piperacillin, and cefuroxime. A lower percentage of resistance (50%) was found to ciprofloxacin and TMP/SMX, as shown in Table 8.

**Table 8:** Antibiotic resistance in Gram-positive isolates

	Frequency	Ampicillin	Amoxicillin-clavulanate	Piperacillin	Pip/Tazo	Imipenem	Meropenem	Cefuroxime	Cefotaxime	Ceftriaxone	Ceftazidime	Ciprofloxacin	Levofloxacin	Moxifloxacin
Gram-positive bacteria	34													
*Lactobacilli*	3	1 (50)	1 (50)	–	0	0	0	–	0	1 (33.3)	–	0	0	–
*Corynebacteria*	2	1 (50)	0	–	0	0	0	–	0	0	0	0	0	–
*E. fecalis*	9	3 (33.3)	3 (33.3)	2 (66.7)	3 (42.9)	–	–	–	–	–	–	3 (37.5)	3 (42.9)	–
*MRSA*	1	–	1 (100)	–	–	–	–	1 (100)	–	–	–	1 (100)	–	0
*MSSA*	1	–	0	–	–	–	–	0	–	–	–	0	0	0
*S. epidermidis*	2	–	2 (100)	2 (100)	2 (100)	–	–	1 (100)	–	–	–	1 (50)	0	0
*S. hemolyticus*	1	–	1 (100)	1 (100)	1 (100)	–	–	1 (100)	–	–	–	1 (100)	1 (100)	0
*S. cohnii*	1	–	1 (100)	–	–	–	–	1 (100)	–	–	–	0	0	0
*S. capitis*	1	–	0	–	–	–	–	0	–	–	–	0	0	0
*S. oralis*	2	–	–	–	–	–	–	–	0	0	–	–	2 (100)	1 (100)

TMP/SMX	Gentamicin	Streptomycin	Amikacin	Benzylpenicillin	Oxacillin	Erythromycin	Quinupristin/Dalfopristin	Vancomycin	Tetracycline	Tigecycline	Nitrofurantoin	Linezolid	Clindamycin	Rifampicin
1 (100)	0	–	0	–	–	0	–	0	0	–	–	–	0	1 (100)
1 (100)	–	–	0	–	–	0	–	0	–	–	–	–	–	–
–	3 (33.3)	3 (33.3)	–	3 (33.3)	–	4 (100)	6 (66.7)	2 (22.2)	4 (50)	0	0	0	–	–
0	0	–	–	1 (100)	1 (100)	1 (100)	0	0	0	0	–	0	0	0
0	0	–	–	1 (100)	0	0	0	0	0	0	–	0	0	0
1 (50)	0	–	–	2 (100)	2 (100)	1 (50)	0	0	1 (50)	0	–	0	0	0
1 (100)	1 (100)	–	–	1 (100)	1 (100)	0	0	0	0	0	–	0	0	0
0	0	–	–	1 (100)	1 (100)	1 (100)	0	0	0	0	–	0	1 (100)	0
0	0	–	–	0	0	0	0	0	0	0	–	0	0	0
–	–	–	–	–	–	2 (100)	–	0	2 (100)	0	–	0	1 (50)	–

*E.fecalis, Enterococcus faecalis*; MRSA, methicillin–resistant *Staphylococcus aureus*; MSSA, methicillin–sensitive *Staphylococcus aureus*; *S. epidermidis*; *Staphylococcus epidermidis*; *S. hemolyticus, Staphylococcus hemolyticus*; *S. cohnii*; *Staphylococcus cohnii*; *S. capitis, Staphylococcus capitis*; *S. oralis, Streptococcus oralis*

### Antimicrobial resistance of Gram-negative isolates

During our study period, 22 isolates of gram-negative bacteria were included. *K. pneumonia* accounted for 7.9% of the total gram-negative bacteria. About 20% of the reported *K. pneumonia* were resistant. Additionally, 40% were resistant to cefepime, and the same for TMP/SMX. A 60% higher resistance of isolated *K. pneumonia* was found to piperacillin/tazobactam, ceftazidime, and ciprofloxacin. Resistance to amoxicillin/clavulanate and ampicillin is high, with 75% and 100%, respectively, among *ESBL-E. Coli*, 66.7%, were resistant to TMP/SMX and ciprofloxacin, while 33% were resistant to piperacillin/tazobactam. All isolates were found to be sensitive. On the other hand, *non-ESBL E. coli* does not show resistance to any of the antibiotics we included in our study.

Unfortunately, the *A. baumannii* isolates were resistant to all tested antibiotics, as shown in Table 9.

**Table 9:** Antibiotic resistance in Gram–negative isolates

	Frequency	Ampicillin	Amoxicillin–clavulanate	Piperacillin	Piperacillin tazobactam	Imipenem	Meropenem	Ertapenem	Cefotaxime	Cefepime
*Gram-negative bacteria*	22	Resistant isolates (% of tested)								
*Klebsiella pneumoniae*	5	5 (100)	3 (75)	–	3 (60)	1 (20)	1 (20)	1 (20)	3 (60)	2 (40)
*ESBL-E. coli*	3	3 (100)	2 (66.7)	–	1 (33.3)	0	0	0	3 (100)	3 (100)
*Non-ESBL E. coli*	2	0	0	–	0	0	0	0	0	0
*Neisseria sicca*	2	–	–	–	0	–	–	–	0	–
*Pseudomonas aeruginosa*	4	–	1 (100)	2 (66.7)	1 (25)	3 (75)	2 (66.7)	–	1 (100)	2 (50)
*Sphingomonas paucimobilis*	1	–	–	–	–	0	0	–	0	0
*E. fergusonii*	1	1 (100)	0	–	0	0	0	0	0	0
*Acinetobacter baumannii*	3	–	1 (100)	3 (100)	–	3 (100)	3 (100)	–	–	3 (100)
*Pseudomonas stutzeri*	1	–	–	0	0	0	0	–	–	0

	Ceftriaxone	Ceftazidime	Ciprofloxacin	TMP/SMX	Gentamicin	Tobramycin	Amikacin	Tetracycline	Minocycline	Nitrofurantoin
*Gram-negative bacteria*	Resistant isolates (% of tested)									
*Klebsiella pneumoniae*	2 (50)	3 (60)	3 (60)	2 (40)	0	–	1 (20)	–	–	–
*ESBL-E. coli*	3 (100)	2 (66.7)	2 (66.7)	2 (66.7)	0	–	0	–	–	–
*Non-ESBL E. coli*	0	0	0	0	0	–	0	–	–	0
*Neisseria sicca*	0	0	–	0	0	–	–	0	–	–
*Pseudomonas aeruginosa*	–	2 (50)	2 (50)	–	2 (66.7)	2 (66.7)	2 (50)	–	–	–
*Sphingomonas paucimobilis*	–	0	0	0	0	–	0	–	–	–
*E. fergusonii*	–	0	1 (100)	1 (100)	0	–	–	–	–	–
*Acinetobacter baumannii*	–	3 (100)	3 (100)	3 (100)	3 (100)	3 (100)	–	–	0	–
*Pseudomonas stutzeri*	–	–	0	0	0	0	0	–	0	–

ESBL, *extended-spectrum beta-lactamase*; *E.* fergusonii***,**** Escherichia fergusoni*

### Antimicrobial resistance in fungal isolates

Two fungal species were isolated throughout our study; one was *C. tropicalis,* which was found to be sensitive to caspofungin, fluconazole, flucytosine, voriconazole, and micafungin. The other isolate was *C. glabrata,* which was also sensitive to all tested antifungal agents.

## Discussion

This study was conducted at a NNUH in Palestine to describe the epidemiology and sources of infection in febrile neutropenic patients. The causes of neutropenic fever can be of infectious or non-infectious origin. The infectious causes of FN can be either microbiologically or clinically documented types. The microbiologically based FN has an identified causative agent, while in the clinically documented FN the microbiologic workup is negative, but there is a high clinical suspicion for infection based on physical exam findings or radiological testing. Approximately 30–50% of the time during the investigation of FN can an infectious origin be determined either microbiologically or clinically [21].

This could be due to an incomplete medical exam or an untimely collection of clinical specimens due to concurrent thrombocytopenia. These patients respond well to empiric antibiotic therapy suggesting a hidden infection. On the other hand, the non-infectious causes of fever may include chemotherapy-induced mucositis, tumor fever, transfusion-related fever, drug-induced fever, or graft-versus-host disease [22].

In our study, 72% of cases of FN were of non-infectious origin, similar to a report from the Kingdom of Saudi Arabia, where Al-Tawfiq et al. (2019) reported a rate of 72.5% [23].

Hematological malignancies affected 86 percent of patients with FN, with acute leukemia accounting for 47% of cases. In contrast, a study from India reported FN episodes were more common in patients with solid tumors (57%) than in those with hematological malignancies, and they were more frequently associated with infections with gram-negative bacteria (56.25%) [24].

We demonstrated an overall mortality of 6.3%, which is low compared to a report from Thailand in 2021 that reported a 12.5% mortality rate in a similar study [25]. Among the infectious causes as the origin of fever, gram-positive bacteria and gram-negative bacteria were found in 54% and 35% respectively. Fungal infections were less common and were found in 3.2% of the cases. Fungal species isolated from our patients included *Candida tropicalis* (1.6%) and *Candida glabrata* (1.6%). Usually in neutropenic patients with cancer, *Aspergillus* spp. and *Candida* spp. are the most frequently isolated organisms, and due to the continuous use of fluconazole as a prophylactic therapy, non- *C. albicans* strains are becoming more prevalent. Prolonged and severe neutropenia is the biggest risk factor for mold infection [21]. Cancer patients are more likely to be infected with gram-negative bacteria [26]. Multidrug-resistant (MDR) gram-negative bacteria are now more frequently responsible for FN-related infections, which makes choosing an antibiotic treatment plan more difficult. Additionally, prolonged empirical antibiotic use has caused a shift in the causative bacteria from gram-negative to gram-positive bacteria. Even though gram-positive bacteria are currently slightly more frequent in infections associated with FN, gram-negative bacteria still make up 40% to 50% of the pathogens found in patients with FN [27]. Infections caused by gram-negative bacteria also have a worse prognosis than infections caused by gram-positive bacteria, with mortality rates of 18% compared to 5% in gram-negative bacteremia [28].

The shift in the bacterial spectrum has raised the issue of whether to alter treatment schedules for proper patient care. However, it has been suggested that epidemiology and local resistance patterns should be considered when selecting empirical antibiotic treatments, as the most efficient treatment approach may vary in various local settings. Numerous related studies have addressed using empirical antibiotic therapy in FN [29–31].

Even though *Staphylococcus* species were cited as the most prevalent gram-positive pathogen in earlier studies [32], the most common gram-positive pathogen in this study was *E. fecalis*. Guidelines for the empirical treatment of FN emphasize the need of *P. aeruginosa* and MRSA coverage. Our study showed that the most frequent gram-positive organism was *Enterococcus* spp. rather than *Staphylococcus* spp. One of the expected findings was that not every isolated pathogen causes infections, such as *S. epidermidis* in blood cultures. This raises concerns about the possibility of contamination because infection with these organisms typically occurs on the skin rather than in the bloodstream [18].

The incidence of *C. difficile* infection was evident in 9.5% of cases and this is higher than what Siddiqi et al. (2019) reported a national incidence of 5.83% [33]. Among the viral causes, COVID-19 constituted 6.3% of febrile neutropenic cases. There are three parts to the antimicrobial prophylaxis strategy: antibacterial, antifungal, and antiviral part [34]. Prophylactic antibiotics used in our study are mostly levofloxacin, acyclovir, and fluconazole. Other regimens that were mentioned in another study were gentamicin, vancomycin, and nystatin [35].

In our study, approximately two-thirds of patients had a MASCC score less than 21, i.e., they were at higher risk of developing complications and should receive immediate treatment with a broad-spectrum parenteral antibiotic. Concerning complications, the most frequent complication was kidney injury (16.7%), followed by the need for oxygen therapy (13.3%), shock (9.3%), and the need for mechanical ventilation (5.3%). Compared to a FINITE study conducted in the US, which included 152 patients, the most frequent complications were shock (7%), acute respiratory failure (6%), and acute kidney injury (3%) [10].

During our study period, 22 isolates of gram-negative bacteria were isolated, five isolates were *K. pneumonia*, and 20% were resistant to ertapenem. In addition, 40% were resistant to cefepime and TMP/SMX. Furthermore, a 50% higher resistance to ceftriaxone was found. Additionally, 60% of isolated *Klebsiella pneumonia* were resistant to piperacillin/tazobactam, cefotaxime, ceftazidime, and ciprofloxacin. Resistance to amoxicillin/clavulanate and ampicillin is high at 75%, and 100%, respectively. Compared to a study conducted in Taiwan, 11 (13%) of 87 K*. pneumoniae* isolates from neutropenic patients were resistant to the cefotaxime, 13% were resistant to ciprofloxacin, 6% were resistant to piperacillin/tazobactam [36].

Regarding *P. aeruginosa*, 100% were resistant to amoxicillin/clavulanate and cefotaxime, 75% were resistant to imipenem, 66.7% were resistant to meropenem and piperacillin and 50% were resistant to ciprofloxacin, ceftazidime, and cefepime. While in the Taiwan study, the great majority of current antibiotics—ciprofloxacin, ceftazidime, cefepime, meropenem, and piperacillin (> 90%) retained good activity against *P. aeruginosa* isolates [36].

Among ESBL-*E. Coli*, 100% were resistant to ampicillin, cefotaxime, cefepime, and ceftriaxone. No isolates were resistant to imipenem, meropenem, or ertapenem. However, in the Taiwan study, 33% were resistant to ciprofloxacin, 10% were resistant to cefotaxime, and no isolates were resistant to imipenem [36].

*E. faecalis* isolates showed high resistance rates to piperacillin (66.7%) and piperacillin/tazobactam (42.9%). Moderate resistance was shown against ciprofloxacin 37.5%) and ampicillin (33.3%).

## Strengths and limitations

This paper is the first in Palestine to study the topic of FN in cancer patients. However, our study has several limitations. First, our data were collected from one center and may not represent other centers. Second, our study is retrospective. Finally, our sample size was relatively small, which could diminish the power of the study.

## Conclusions

Febrile neutropenia is a life-threatening complication that increases morbidity and mortality in cancer patients. Furthermore, the present study suggests that many patients developed kidney injury and shock. In contrast to previous international studies, gram-positive bacteria are also the most common cause of FN than gram-negative bacteria. *Enterococcus feacalis* were the most common among gram-positive isolates. As for gram-negative*, klebsiella pneumonia* and *E. coli* were the most common. This study is the first in Palestine to study the topic of FN in cancer patients.

## Data Availability

The data from our surveillance are not available on the public domain due to privacy and ethical restrictions, but anyone interested in using the data for scientific purposes is free to request permission from the corresponding authors.

## Abbreviations

ALL: Acute lymphoid leukemia
AML: Acute myelogenous leukemia
BSI: Bloodstream infection
CHF: Congestive heart failure
CLL: Chronic lymphoid leukemia
CoNS: Coagulase-negative Staphylococci
COPD: Chronic obstructive pulmonary disease
COVID: Coronavirus Disease
CVC: Central venous catheters
ESBL: Extended Spectrum Beta-Lactamase
FN: Febrile neutropenia
HTN: Hypertension
ICU: Intensive care unit
IQR: Interquartile range
MASCC: Multinational Association of Supportive Care of Cancer
MRSA: Methicillin-Resistant *Staphylococcus* *aureus*
MSSA: Methicillin-sensitive *Staphylococcus* *aureus*
SD: Standard deviation
TMP/SMX: Trimethoprim/Sulfamethoxazole
PCOS: Polycystic Ovarian Syndrome
VRE: Vancomycin-resistance Enterococcus
